# Undiagnosed Metachromatic Leukodystrophy Presenting as Severe Gastrointestinal Bleeding and Cholestasis from Hemobilia

**DOI:** 10.1097/PG9.0000000000000122

**Published:** 2021-09-23

**Authors:** James P. Stevens, Janani Dakshinamoorthy, Anne Elizabeth Gill, Paul Parker, Duke Geem, John-Paul Berauer, Bess Schoen, Nitika Gupta, Rene Romero

**Affiliations:** From the * Division of Gastroenterology, Hepatology, and Nutrition, Children’s Healthcare of Atlanta, Atlanta, GA; † Department of Pediatrics, Emory University School of Medicine, Atlanta, GA; ‡ Department of Radiology & Imaging Sciences, Division of Interventional Radiology and Image-Guided Medicine, Emory University School of Medicine, Atlanta, GA; § Division of Pediatric Surgery, Department of Surgery, Emory University School of Medicine, Atlanta, GA.

**Keywords:** metachromatic leukodystrophy, hemobilia, upper gastrointestinal bleeding, gallbladder polyposis

## Abstract

Metachromatic leukodystrophy (MLD) is a neurodegenerative disorder caused by the accumulation of lipids called sulfatides throughout the nervous system. Sulfatides can also collect in other organs throughout the body including the gallbladder where they form polyps. Gallbladder polyps rarely have been found to bleed in patients with known MLD, presumably due to polyp shearing. Here we present a case of a child with autism presenting with severe gastrointestinal bleeding and direct hyperbilirubinemia, requiring significant resuscitation and biliary drain placement to tamponade ongoing bleeding. Subsequent neurologic and genetic investigation led to the diagnosis of MLD, with laparoscopic cholecystectomy revealing extensive, elongated gallbladder polyps. Clinicians who care for patients with MLD, including gastroenterologists who manage their progressive oropharyngeal dysphagia, should be aware of the risk for this life-threatening complication. Moreover, pediatric gastroenterologists and hepatologists should maintain a high index of suspicion for MLD in new patients presenting with developmental regression and gastrointestinal bleeding.

## INTRODUCTION

Metachromatic Leukodystrophy (MLD) is a rare, progressive, neurodegenerative demyelinating disorder that presents in different forms from infancy to adulthood. It is an autosomal recessive, inherited disease characterized by the accumulation of sulfatides (glycolipids) in the central and peripheral nervous systems. Sulfatides have also been shown to collect in other organs of MLD patients, including forming polyps within the gallbladder (GB) (1–4). We describe the case of a child presenting with severe gastrointestinal bleeding from hemobilia, found to have extensive gallbladder polyposis and with concurrent hospital investigation leading to the new diagnosis of MLD.

## CASE REPORT

A 5-year-old male with autism spectrum disorder presented to the emergency department with acute hematemesis and melena. He was tachycardic with initial laboratory investigation notable for anemia (hemoglobin 6.9 g/dL), elevated transaminases (aspartate aminotransferase 310 U/L, alanine aminotranferase 510 U/L), direct hyperbilirubinemia (total bilirubin 3.6 mg/dL, direct 2.7 mg/dL), and elevated gamma-glutamyl transferase (325 U/L). Synthetic liver function was intact (prothrombin time 15.1 seconds; international normalized ratio 1.2). Abdominal ultrasound (US) showed GB wall thickening to 6.5 mm, GB sludge, and common bile duct (CBD) dilatation to 4 mm, with sludge in the CBD and common hepatic duct (CHD).

The patient had ongoing bleeding, in total requiring six units packed red blood cells, two units fresh frozen plasma, and one unit of platelets. Urgent esophagogastroduodenoscopy (EGD) was performed hospital day (HD) no. 1, showing normal esophageal and gastric mucosa. A blood clot obstructed visualization of the ampulla of Vater, with blood throughout the duodenum but no identifiable area of active bleeding from the duodenal mucosa that was normal in appearance. No intervention was performed during EGD. Endoscopic retrograde cholangiopancreatography (ERCP) was not done during EGD due to the large clot near the ampulla with overall poor visualization, without a confirmed specific source of the bleeding, and given that our ERCP-trained endoscopist was not readily available at the time of the procedure.

**FIGURE 1.**
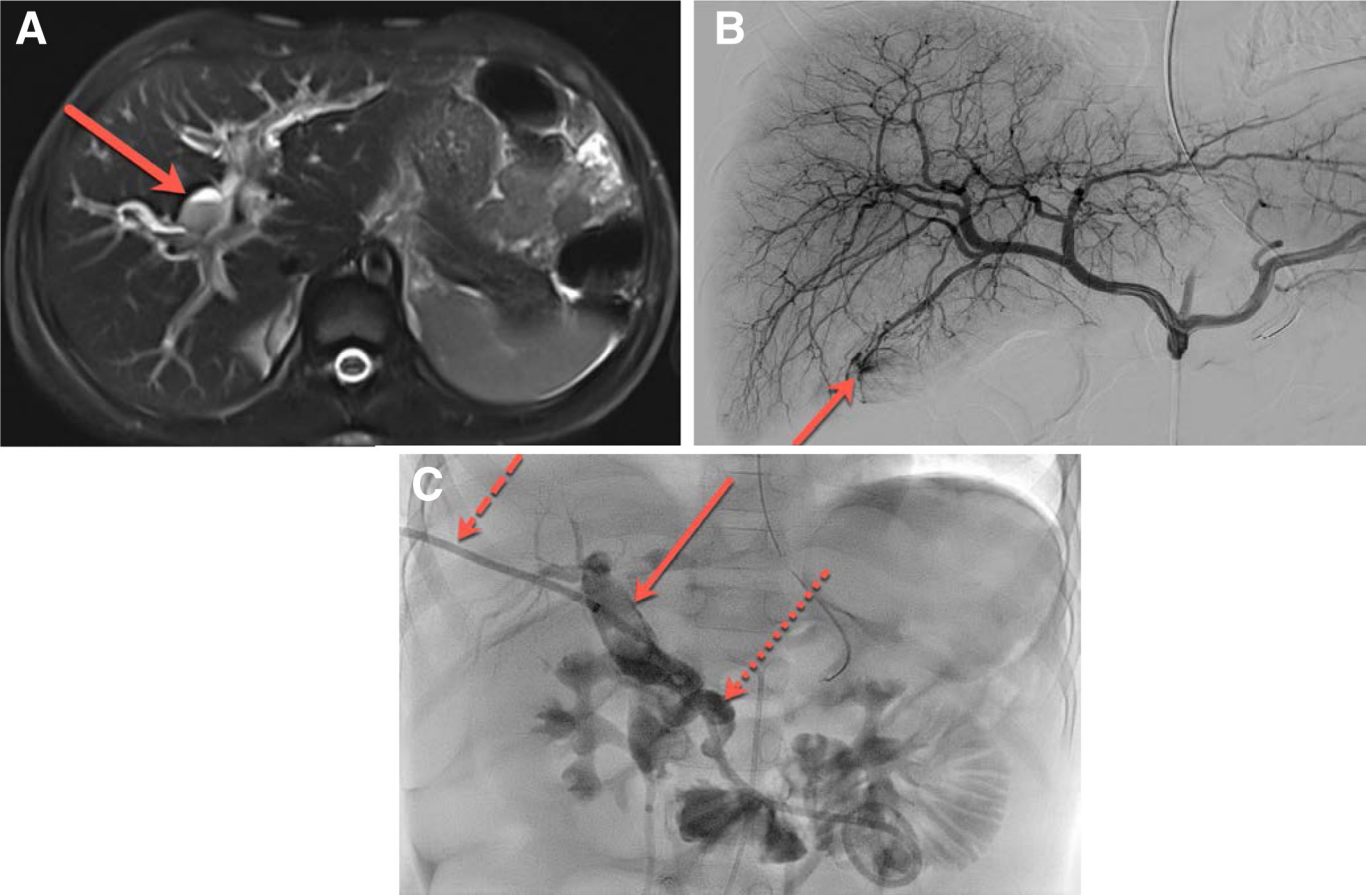
A) Axial T2 weighted sequence of MRCP shows dilated biliary structures with surrounding edema. Central dilation of biliary structures was thought to represent choledochal cyst. Within the dilated central biliary structure there is a fluid-fluid layer (red arrow), which shows layering debris (found to represent blood products) and bile (nondependent fluid). B) Digital subtraction angiography from the proper hepatic artery shows a conventional branching pattern of the right and left hepatic artery as well as hyperemia and slight blush of contrast at the distal tip of the cystic artery, supplying the gallbladder (red arrow). C) Cholangiogram through the transhepatic catheter (dashed red arrow) shows markedly dilated central biliary structures with superimposed clot burden (solid red arrow). Additionally, there is a tortuous and dilated course of the common bile duct (dotted red arrow). Catheter tip terminates in loops of small bowel. MRCP = magnetic resonance cholangiopancreatography.

Magnetic resonance cholangiopancreatography (MRCP) performed on HD no. 2 confirmed US findings in addition to cystic dilatation of the extrahepatic CHD up to 10 mm. This suggested a possible type 4B choledochal cyst (Fig. 1A) with an infected cyst eroding into the common hepatic artery (CHA). Due to the concern for possible bleeding CHA, diagnostic angiography was performed, with the option of embolization if needed. Neither angiography nor later computed tomography angiogram revealed a source of bleeding requiring intervention, although angiography noted significant hyperemia at the tip of the gallbladder (Fig. 1B).

Percutaneous transhepatic cholangiogram (PTC) was performed concurrently with angiography, with internal/external biliary drain placed to tamponade bleeding (Fig. 1C). We elected for PTC instead of ERCP, as we could combine angiography and PTC into one procedure performed by our highly experienced interventional radiologists. Biliary drain output was initially bloody but turned bilious over 72 hours. The patient’s bilirubin and transaminases rapidly improved after drain placement. Hemoglobin stabilized by HD no. 5, and melena resolved.

**FIGURE 2.**
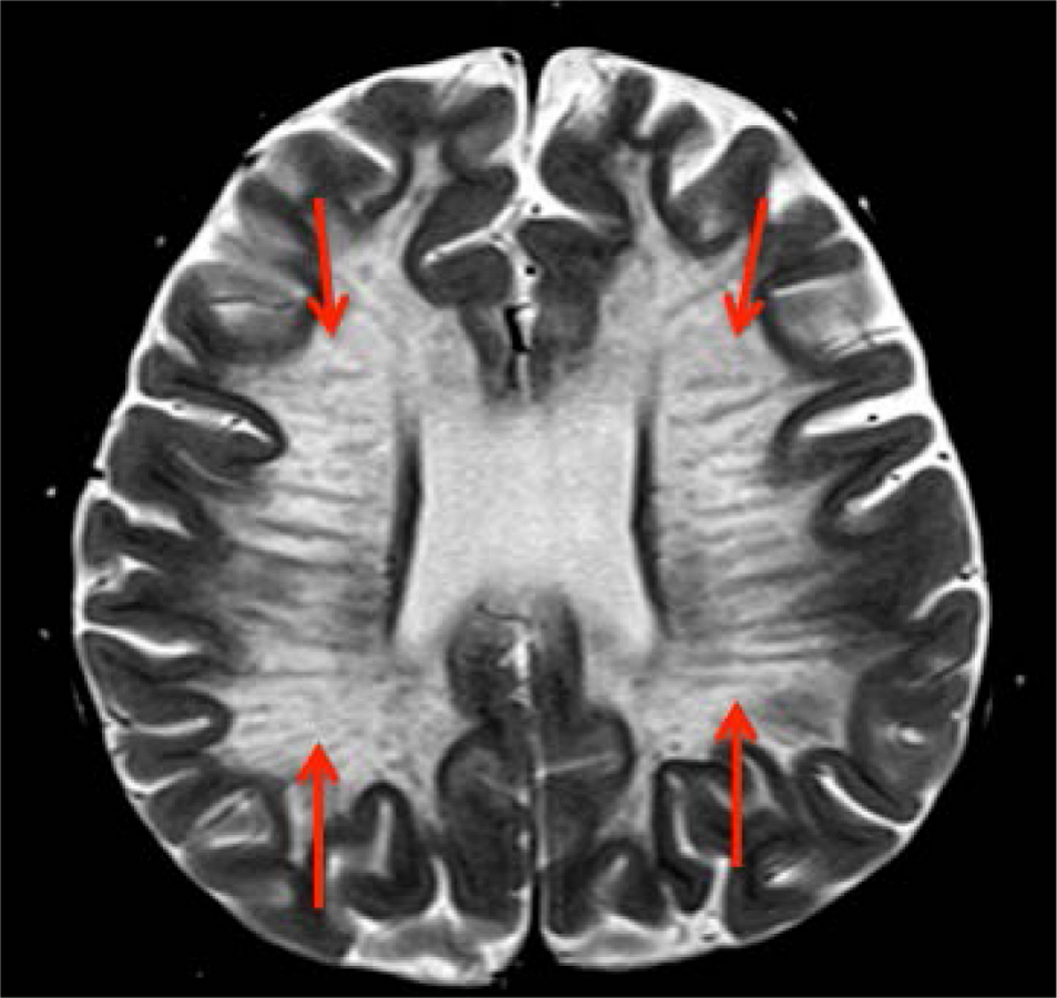
Magnetic resonance imaging of patient’s brain showing classic “Tigroid” or “Leopard Skin” pattern in the periventricular white matter on T2W images, consistent with metachromatic leukodystrophy (red arrows).

Additional history obtained during the admission revealed the patient had normal development for the first 4 years of life, but over the last 4 months lost several gross motor, fine motor, and language skills previously attained. His pediatrician gave him a presumed diagnosis of autism, although no formal evaluation was completed. Pediatric neurology and medical genetics were consulted inpatient, and an MRI brain was performed, which was consistent with MLD (Fig. 2). Genetic testing revealed two heterozygous variants in the arylsulfatase A (*ARSA*) gene associated with MLD: one pathogenic (*p.Glu309Lys*) and one of unknown significance (*p.Val388Phe*). His urine also resulted positive for sulfatides.

**FIGURE 3.**
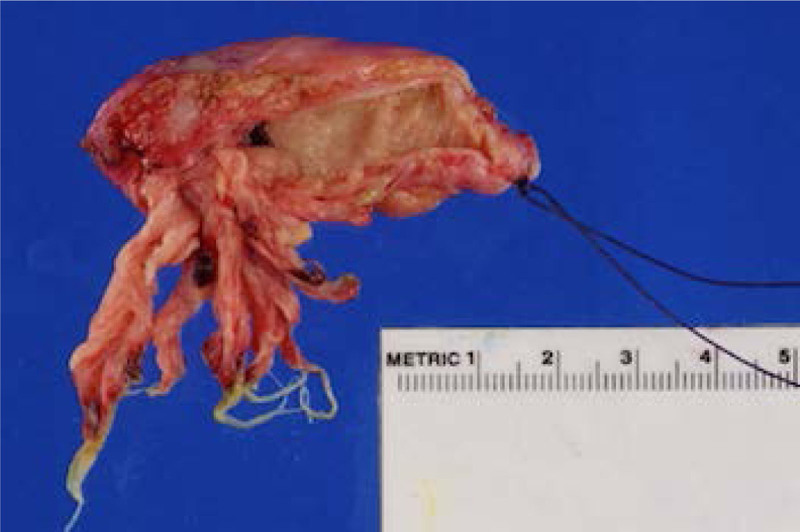
Photograph of patient’s gallbladder with extensive polyposis.

On HD no. 15, he underwent laparotomy, cholecystectomy, and intraoperative cholangiogram (IOC). The GB wall was thickened, with long tentacle-like growths throughout and evidence of old blood (Fig. 3). GB histology revealed columnar mucinous papillary proliferation and goblet cells reflecting intestinal metaplasia, with increased histiocytes in the lamina propria consistent with MLD. Electron microscopy displayed lysosomal inclusions with herringbone and prismatic patterns characteristic of sulfatide accumulation. IOC showed a now normal size CBD, but persistent dilatation of CHD with fusiform filling. Given the patient’s reduced likelihood of survival into adulthood, the possible CHD cyst was not surgically removed. The patient recovered, biliary drain was removed and he was discharged with appropriate subspecialty follow-up.

Repeat ultrasound 2 months after discharge showed no evidence of choledochal cyst or biliary dilation. He continues to follow in our multidisciplinary leukodystrophy clinic and in our gastroenterology clinic for progressive oropharyngeal dysphagia associated with his neurodegenerative disorder.

## DISCUSSION

Our case provides insight into a rare, life-threatening gastrointestinal complication of a neurodegenerative disorder. Only a few prior cases of a child or adult with known MLD presenting with anemia from hemobilia are reported (5–7).

Bleeding is presumed to be due to the shearing of GB polyps, with no other source of bleeding identified in our patient. We were unable to find any prior cases in which a reportedly healthy child presented with massive upper gastrointestinal bleeding, which led to the new diagnosis of MLD. GB polyposis in MLD can also present as abdominal pain, vomiting, gastric distention, and poor oral intake (1, 3). Ries et al described a patient who developed chronic pancreatitis with pseudocyst formation prior to the onset of any neurologic sequelae (2).

Gallbladder polyps are extremely uncommon in pediatrics but are well documented in MLD, Peutz-Jeghers, and Gardner syndrome (8). GB polyposis can be familial outside of these specific syndromes, and conditions such as hepatitis B infection, recurrent cholecystitis, hypercholesterolemia, and metabolic syndrome can increase risk of polyp formation (9). Van Rappard et al found GB polyps on US in 23% of patients with MLD, while any GB abnormality was found in 77% (often GB wall thickening, gallstones, or sludge) (4). GB findings can vary significantly even between siblings with MLD (3). Histological findings include columnar mucinous papillary projections with gastric or intestinal metaplasia, containing pigment-laden “metachromatic” macrophages within the polyps (1). Rokitansky-Aschoff sinuses (diverticula) can be seen within the gallbladder (1). Malignant transformation is a concern, with GB adenocarcinomas documented in adults with MLD (4).

Patients with MLD often develop oropharyngeal dysphagia due to the direct neurodegenerative effects of their disease. They can have increasing weakness, poor oropharyngeal coordination, and deficits in their swallowing which put them at risk for both anterior loss of food and aspiration, as was the case for our patient. As their disease progresses, many patients become dependent on gastrostomy tube feeds for their nutrition and thus may present to a pediatric gastroenterology clinic for poor weight gain or feeding tube management. In these patients, one must consider assessing the risk of GB polyposis and bleeding as part of management.

No formal guidelines exist on the role of US in asymptomatic MLD patients. Van Rappard et al recommended screening all patients and performing laparoscopic cholecystectomies on those with GB thickening or with polyps >5 mm (4). Cholecystectomy could prevent both malignancy and life-threatening hemobilia. However, the decision to perform cholecystectomy and its timing in a patient with a life-limiting disease must be carefully considered.

In summary, gallbladder polyposis in MLD can lead to life-threatening complications such as massive hemobilia. Providers caring for patients with MLD should be aware of the possible presentations of polyposis and can consider imaging both symptomatic and asymptomatic patients. MLD should be within the differential for any patient presenting with developmental regression and gastrointestinal bleeding.
